# Safety assessment of laronidase: real-world adverse event analysis based on the FDA adverse event reporting system (FAERS)

**DOI:** 10.3389/fphar.2025.1623921

**Published:** 2025-08-20

**Authors:** Zhuomiao Lin, Junling Xue, Meiqing Yang, Xihui Yu, Jiahong Zhong

**Affiliations:** ^1^ Department of Clinical Pharmacy, Meizhou People’s Hospital (Huangtang Hospital), Meizhou, China; ^2^ Department of Pharmacy, The Second Affiliated Hospital of Shantou University Medical College, Shantou, China; ^3^ Joint Shantou International Eye Center, Shantou University and the Chinese University of Hong Kong, Shantou, China

**Keywords:** laronidase, FAERS, adverse events, mucopolysaccharidosis type I, pharmacovigilance

## Abstract

**Objective:**

Laronidase is the first drug of enzyme replacement therapy approved for the treatment of mucopolysaccharidosis type I (MPS I). However, its adverse events (AEs) have not been investigated in real - world settings. The aim of this study was to investigate AEs associated with laronidase using the Food and Drug Administration Adverse Event Reporting System (FAERS).

**Methods:**

Data for laronidase were acquired from the FAERS database covering Q1 2004 through Q4 2024. The Reporting Odds Ratio (ROR), Proportional Reporting Ratio (PRR), Bayesian Confidence Propagation Neural Network (BCPNN) and Multi-item Gamma Poisson Shrinker (MGPS) were employed to identify potential safety signals.

**Results:**

A total of 3,677 adverse event reports associated with laronidase were identified in the FAERS from 2004 to 2024. The results revealed that common AEs of laronidase such as pyrexia [n = 465, ROR = 6.23 (5.68–6.83)], pneumonia [n = 223, ROR = 3.22 (2.82–3.68)], cough [n = 167, ROR = 2.78 (2.38–3.23)], influenza [n = 114, ROR = 4.95 (4.12–5.95)], urticaria [n = 106, ROR = 2.99 (2.47–3.62)], disease progression [n = 101, ROR = 3.95 (3.25–4.81)]. Furthermore, we detected probable unexpected AEs like seizures [n = 75, ROR = 3.1 (2.47–3.89)], hydrocephalus [n = 60, ROR = 50.47 (39.1–65.14)], blindness [n = 44, ROR = 5.02 (3.73–6.75)], glaucoma [n = 32, ROR = 7.56 (5.34–10.69)]. Laronidase -induced adverse reactions involved 27 System Organ Class (SOC). No significant difference in AEs was observed between sexes for laronidase. Most AEs (n = 763) emerged more than 360 days following laronidase treatment.

**Conclusion:**

Our study has identified AEs associated with laronidase that could provide support for clinical monitoring and risk identification of laronidase.

## 1 Introduction

Mucopolysaccharidosis type I (MPS I) is an autosomal recessive lysosomal disorder caused by mutations in the α-L-iduronidase (*IDUA*) gene, which encodes the enzyme responsible for glycosaminoglycan (GAG) catabolism. Deficient IDUA activity leads to accumulation of undegraded GAGs within lysosomes, triggering multisystemic pathology (Machnikowska-Sokolowska et al., 2023). With an estimated prevalence of 1 per 100,000 live births, this progressive disorder manifests as intellectual disability, short stature, joint stiffness, hepatosplenomegaly, hearing impairment, cardiac valve abnormalities, and corneal clouding (Machnikowska-Sokolowska et al., 2023; Chen et al., 2016). Furthermore, the disease will follow a relentless trajectory without treatment, eventually leading to severe disability and premature death (Machnikowska-Sokolowska et al., 2023). Therefore, early diagnosis and therapeutic intervention are critical to mitigate clinical deterioration, improve patient quality of life, and alleviate socioeconomic burdens. The primary therapeutic strategies for MPS-I are hematopoietic stem cell transplantation (HSCT) and enzyme replacement therapy (ERT), with ERT being applicable as either a standalone treatment or as an adjunctive therapy during HSCT (Lipinski et al., 2024; Hampe et al., 2021a).

Laronidase, a recombinant IDUA enzyme, was approved as a long-term enzyme replacement therapy for MPS I by the US Food and Drug Administration (FDA) in 2003 and by the National Medical Products Administration in China in 2020, respectively (Li et al., 2022; The Medical Letter, 2003). The treatment principle involves administering exogenous enzymes that can be internalized by lysosomes, thereby augmenting GAGs catabolism and preventing their pathological accumulation in tissues (Jameson et al., 2019). In a clinical study involving 45 patients with MPS I aged 6–24 years, weekly intravenous infusions of laronidase administered over a 26-week period resulted in significant reductions in urinary glycosaminoglycan (uGAG) levels, improvements in forced vital capacity, and enhancements in the 6-min walk distance (Clarke et al., 2009). Studies conducted in Italy, Japan, and other regions have consistently reported similar therapeutic outcomes (Pjetraj et al., 2023; Yamazaki et al., 2019). Collectively, laronidase has demonstrated a favorable safety profile and sustained clinical benefits, supporting its role as a reliable therapeutic intervention for MPS I. However, the limited patient population receiving laronidase therapy has raised concerns among clinicians and patients regarding its long-term safety profile. A randomized, open-label, controlled pilot study identified a broad spectrum of adverse events (AEs) associated with laronidase, including injection site pain, back/groin discomfort, headache, cervical muscle spasms, and transient visual disturbances (Chen et al., 2020). These findings underscore the necessity for further investigations to delineate real-world AE signals, identify rare and severe drug-related AEs, and establish robust pharmacovigilance protocols to ensure the safe administration of laronidase in MPS I patients.

The inherent limitations of clinical trials, including underreporting of delayed or infrequent AEs, may obscure comprehensive safety assessments of laronidase in MPS I. Although post-marketing surveillance data could partially mitigate these shortcomings, the evaluation of laronidase remains challenging due to the rarity and complexity of MPS I. The existing clinical trials are subject to the dual limitations of insufficient sample size and inadequate follow-up duration. These constraints impede the identification of rare AEs and hinder a comprehensive assessment of long-term safety, which is particularly crucial for diseases with extremely low prevalence (Pjetraj et al., 2023; Yamazaki et al., 2019; Li et al., 2022). This study utilized the FDA Adverse Event Reporting System (FAERS) to identify potential unreported AEs and assess the long-term safety profile of laronidase in the real-world setting.

## 2 Materials and methods

### 2.1 Data source and collection

We conducted a retrospective pharmacovigilance study using the FAERS database to evaluate AEs associated with laronidase. The FAERS database, a publicly accessible pharmacovigilance platform established in 2004, aggregates de-identified safety reports submitted by global consumers, healthcare professionals, and pharmaceutical manufacturers. Aligned with the post-marketing timeline of laronidase (FDA-approved in 2003), AE data spanning from Q1 2004 to Q4 2024 were extracted for systematic analysis.

### 2.2 Data processing

**FIGURE 1:**
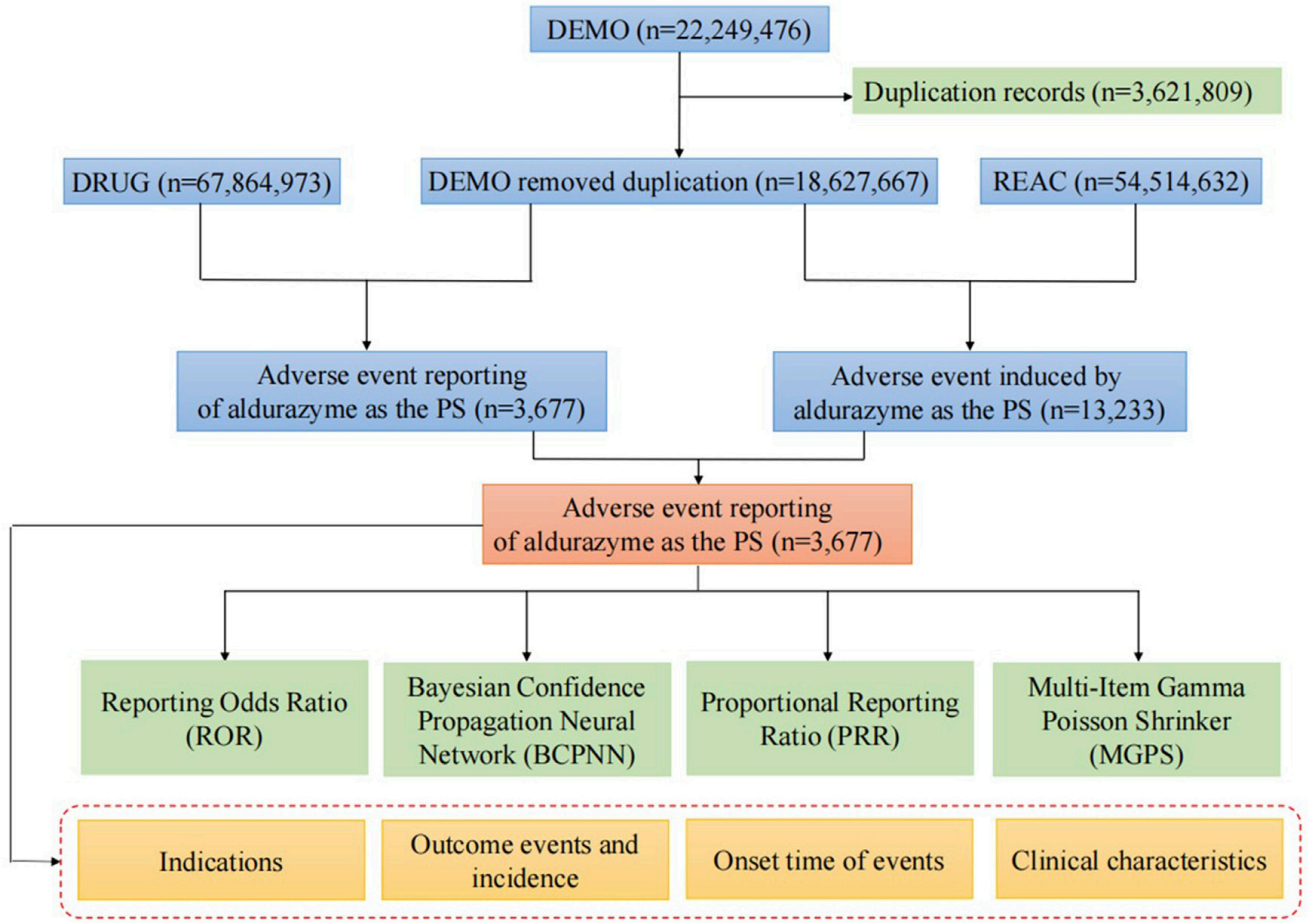
Flow diagram of the study (DEMO, demographic and administrative information; DRUG, drug Information; REAC, preferred terminology for adverse drug reactions; PS, primary suspect drug).

We downloaded the XML data package and imported it into RStudio, following the FDA’s recommendations for data cleaning. To ensure comprehensive capture of all relevant reports, we identified relevant reports using the generic name “laronidase” and the brand name “aldurazyme.” Only reports where laronidase was listed as the primary suspected drug (PS) were selected for analysis. When citing AE names from the reports, we used the Preferred Term (PT) from the Medical Dictionary for Regulatory Activities (MedDRA) for standardized coding. We also classified all AEs according to the System Organ Class (SOC). Duplicate reports occurred when both consumers and sponsors submitted the same report. Following the FDA’s recommended method for removing duplicate reports, we selected the PRIMARYID, CASEID, and FDA_DT fields from the DEMO table. We sorted the data by CASEID, followed by FDA_DT, and then by PRIMARYID. For reports with the same CASEID, we retained the report with the highest FDA_DT value, as a higher value indicates a more recent report date. For reports with identical CASEID and FDA_DT values, we retained the report with the largest PRIMARYID value. After cleaning and standardization, the data was compiled into a final dataset ready for analysis. This dataset included only reports where laronidase was listed as the primary suspected drug, aligning with the focus of our study. During the study period, we obtained 22,249,476 reports related to laronidase from the FAERS database. After excluding duplicates, there were 18,627,667 reports remaining, with 3,677 AEs associated with laronidase (Figure 1). All AE reports related to laronidase were analyzed at the SOC and PT levels.

### 2.3 Statistical analysis

High-sensitivity methods are employed to detect potential AEs with reduced risk of missing true signals, whereas high-specificity approaches mitigate false-positive signal misinterpretation. For example, the Reporting Odds Ratio (ROR) and Proportional Reporting Ratio (PRR) exhibit high sensitivity but low specificity, whereas Bayesian Confidence Propagation Neural Network (BCPNN) and Multi-Item Gamma Poisson Shrinker (MGPS) prioritize specificity at the expense of sensitivity (Liu et al., 2025). In this study, we selected ROR and PRR methodologies to maximize the identification of underreported AE signals. To further address potential confounding from false positives, BCPNN was chosen over other specificity-focused methods due to its enhanced performance in scenarios with elevated confounding factors or larger effect sizes. The computational formulas and decision thresholds for these four algorithms are detailed in Table 1. Data processing and statistical analyses were performed using Microsoft Excel 2021 and R software (version 4.3.0).

**TABLE 1:** Calculation formula and standard of signal detection.

Algorithm	Calculation formula	Criterion
ROR	$\text{ROR}=\frac{a/c}{b/d}=\frac{ad}{bc}$	a ≥3
	$95\%\text{CI}=e^{\ln\left(ROR\right)\pm 1.96\sqrt{\frac{1}{a}+\frac{1}{b}+\frac{1}{c}+\frac{1}{d}}}$	95%CI (lower limit) > 1
PRR	$\text{PRR}=\frac{a/\left(a+b\right)}{c/\left(c+d\right)}$	a ≥3, PRR ≥2
	$\chi^{2}=\frac{{\left(ad-bc\right)}^{2}\left(a+b+c+d\right)}{\left(a+b\right)\left(a+c\right)\left(c+d\right)\left(b+d\right)}$	χ^2^ ≥ 4
EBGM	$\text{EGBM}=\frac{a\left(a+b+c+d\right)}{\left(a+b\right)\left(a+c\right)}$	EBGM05 > 2
	$\text{EBGM}05=e^{\ln\left(EBGM\right)-1.64\sqrt{\frac{1}{a}+\frac{1}{b}+\frac{1}{c}+\frac{1}{d}}}$	
BCPNN	$\text{IC}=\log_{2}\frac{a\left(a+b+c+d\right)}{\left(a+b\right)\left(a+c\right)}$	a ≥3
	$E\left(IC\right)=\log_{2}\frac{\left(a+\gamma_{11}\right)\left(N+\alpha \right)\left(N+\beta \right)}{\left(N+\gamma \right)\left(a+b+\alpha_{1}\right)\left(a+c+\beta_{1}\right)}$ $V\left(IC\right)=\frac{1}{{\left(\ln2\right)}^{2}}\left[\frac{N-a+\gamma -\gamma_{11}}{\left(a+\gamma_{11}\right)\left(N+1+\gamma \right)}+\frac{N-a-b+\alpha -\alpha_{1}}{\left(a+b+\alpha_{1}\right)\left(N+1+\alpha \right)}+\frac{N-a-c+\beta -\beta_{1}}{\left(a+c+\beta_{1}\right)\left(N+1+\beta \right)}\right]$ $\gamma =\gamma_{11}\frac{\left(N+\alpha \right)\left(N+\beta \right)}{\left(a+b+\alpha_{1}\right)\left(a+c+\beta_{1}\right)}$ $95\%CI=E\left(IC\right)\pm 1.96\sqrt{V\left(IC\right)}$ Where α = α_1_+α_2_, β = β_1_+β_2_, N = a+b + c + d, and the value of α_1_, α_2_, β_1_, β_2_ and γ_11_ were defined as 1	The lower limit of 95%CI (IC025) > 0

Abbreviations: a, number of reports containing both the target drug and target adverse drug reaction; b, number of reports containing other adverse drug reaction of the target drug; c, number of reports containing the target adverse drug reaction of other drugs; d, number of reports containing other drugs and other adverse drug reactions. 95%CI, 95% confidence interval; χ^2^, chi-squared; IC, information component; IC025, the lower limit of 95% CI of the IC; EBGM, empirical bayesian geometric mean; EBGM05, the lower limit of 90% CI of the EBGM.

## 3 Results

### 3.1 General characteristics

A total of 3,677 AE reports associated with laronidase were identified in the FAERS database from Q1 2004 to Q4 2024. The clinical characteristics of these reports were summarized in Table 2. Sex distribution revealed females accounted for 36.0% of cases and males for 36.3%. While age data were unavailable for most reports, the analyzable subset showed a predominance of patients aged 5–18 years (28.6%), followed by adults over 18 years (16.9%). Geographically, reports predominantly originated from the United States (73.7%). Consumer-submitted reports constituted the majority (65.5%) of cases. After excluding reports with unknown outcomes, serious outcomes (classified as important medical events) represented the most frequently documented AE severity category. Furthermore, death cases account for 18.9%.

**TABLE 2:** Characteristics of reports associated with laronidase from the FAERS database (Q1 2004-Q4 2024).

Factors	Number of events	Events, %
Case reports	3677	100
Gender		
Female	1324	36.0
Male	1334	36.3
Unknown	1019	27.7
Age (year)		
<5	585	15.9
≥5 and ≤18	1052	28.6
≥18	620	16.9
Unknown	1420	38.6
Reporter		
Consumer	2407	65.5
Health Professional	226	6.1
Physician	810	22.0
Pharmacist	51	1.4
Registered Nurse	3	0.1
Unknown	25	0.7
Reporter country		
United States	2710	73.7
Brazil	117	3.2
Others	850	23.1
Outcome		
Hospitalization (initial or prolonged)	1012	27.5
Death	610	16.6
Congenital Anomaly	5	0.1
Life threatening	55	1.5
Disability	69	1.9
Other serious (important medical event)	785	21.3
Unknown	1141	31.0
Reporting year		
2004	81	2.2
2005	60	1.63
2006	28	0.76
2007	110	2.99
2008	58	1.58
2009	56	1.52
2010	50	1.36
2011	43	1.17
2012	69	1.88
2013	131	3.56
2014	127	3.45
2015	180	4.9
2016	201	5.47
2017	236	6.42
2018	295	8.02
2019	263	7.15
2020	274	7.45
2021	315	8.57
2022	387	10.52
2023	366	9.95
2024	347	9.44

### 3.2 Signal detection

**FIGURE 2:**
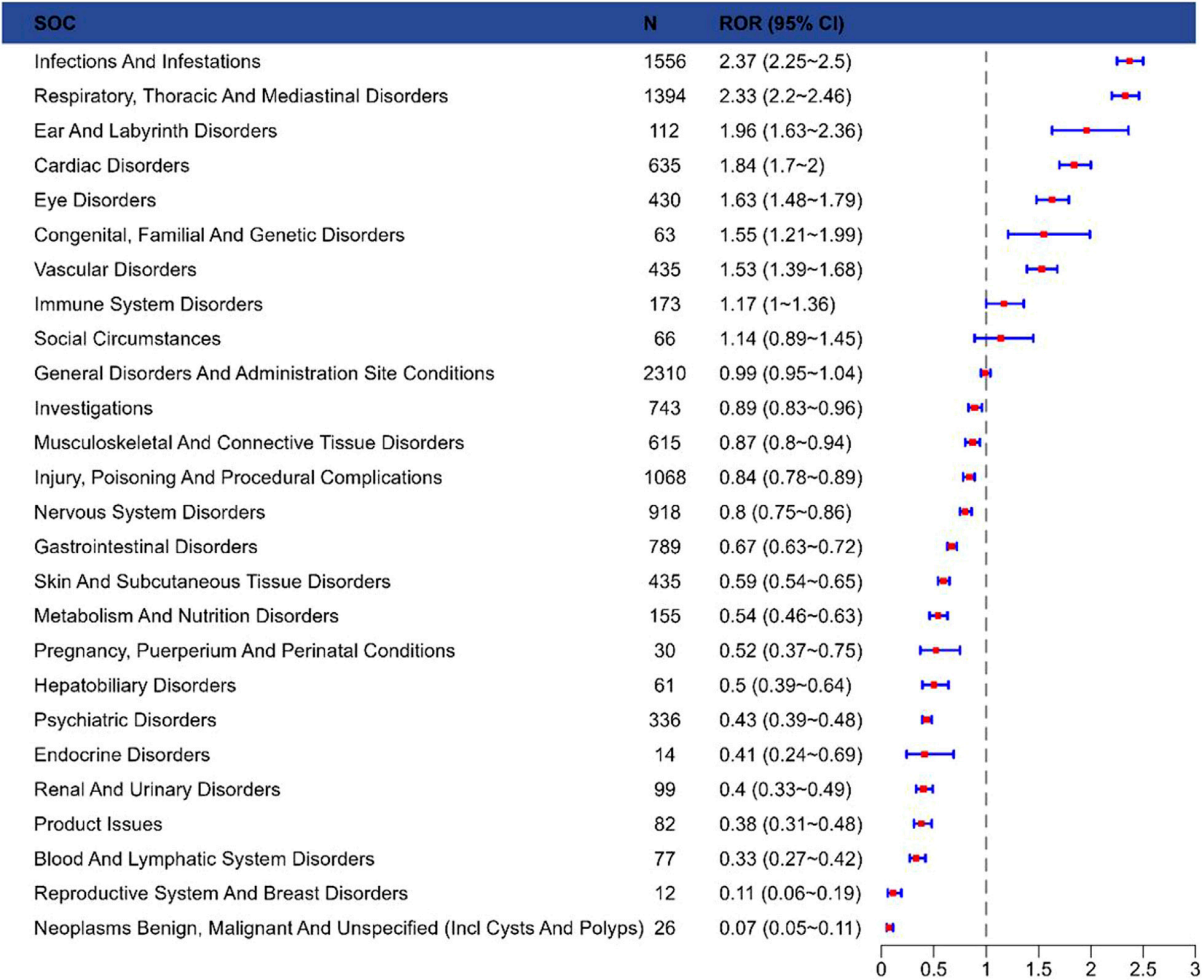
Reporting odds ratios with 95% CI for adverse events at the System Organ Class level. Abbreviations: SOC, system organ class; ROR, reporting odds ratio; Cl, confidence interval.

The signal strength of laronidase across SOCs was summarized in Figure 2 (raw data in Supplementary Table S1). Systematic analysis identified 27 organ systems impacted by laronidase-associated adverse drug reactions. The most frequently reported SOC was infections and infestations [n = 1,556, ROR = 2.37 (2.25–2.5)].

**FIGURE 3:**
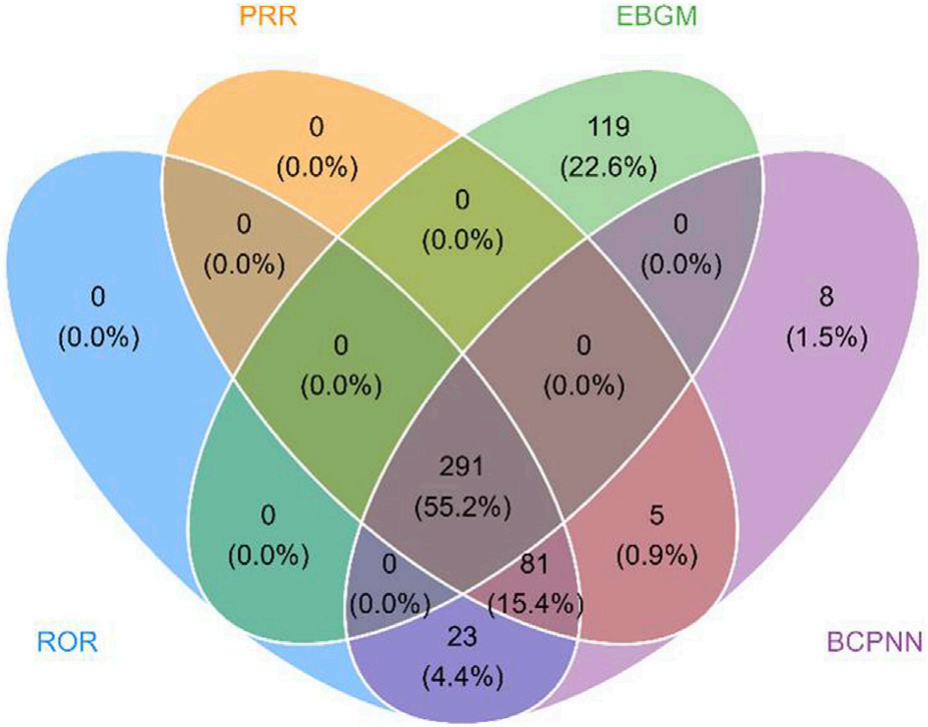
Venn diagram of PT signals meeting the criteria of four algorithms. Abbreviations: PRR, proportional reporting ratio; ROR, reporting odds ratio; EBGM, empirical bayesian geometric mean; BCPNN, bayesian confidence propagation neural network.

The results presented the top 20 PTs ranked by their frequency of occurrence in Table 3. The top 5 most frequently reported PTs in rank order were pyrexia [n = 465, ROR = 6.23 (5.68–6.83), IC025 = 2.59, PRR = 6.05, EBGM05 = 6.04], pneumonia [n = 223, ROR = 3.22 (2.82–3.68), IC025 = 1.67, PRR = 3.18, EBGM05 = 3.18], cough [n = 167, ROR = 2.78 (2.38–3.23), IC025 = 1.46, PRR = 2.75, EBGM05 = 2.75], influenza [n = 114, ROR = 4.95 (4.12–5.95), IC025 = 2.3, PRR = 4.92, EBGM05 = 4.91], urticaria [n = 106, ROR = 2.99 (2.47–3.62), IC025 = 1.57, PRR = 2.98, EBGM05 = 2.97]. In the FAERS database, four algorithms collectively identified 291 PT signals associated with laronidase, spanning 12 SOCs (raw data in Supplementary Table S2). The detailed screening process was illustrated in Figure 3. Notably, among the top 20 PTs by reporting frequency, seizure [n = 75, ROR = 3.1 (2.47–3.89), IC025 = 1.63, PRR = 3.09, EBGM05 = 3.09] and hydrocephalus [n = 60, ROR = 50.47 (39.1–65.14), IC025 = 5.63, PRR = 50.24, EBGM05 = 49.65] were not documented in the drug label.

**TABLE 3:** Top 20 frequency of adverse events at the PT level for laronidase.

SOC	PT (Preferred Term)	Case number	ROR (95%Cl)	IC (IC025)	PRR (χ^2^)	EBGM (EBGM05)
General Disorders And Administration Site Conditions	Pyrexia	465	6.23 (5.68–6.83)	2.59 (2.46)	6.05 (1966.66)	6.04 (5.59)
Infections And Infestations	Pneumonia	223	3.22 (2.82–3.68)	1.67 (1.47)	3.18 (335.25)	3.18 (2.85)
Respiratory, Thoracic And Mediastinal Disorders	Cough	167	2.78 (2.38–3.23)	1.46 (1.24)	2.75 (187.28)	2.75 (2.42)
Infections And Infestations	Influenza	114	4.95 (4.12–5.95)	2.3 (2.03)	4.92 (355.75)	4.91 (4.21)
Skin And Subcutaneous Tissue Disorders	Urticaria	106	2.99 (2.47–3.62)	1.57 (1.29)	2.98 (139.33)	2.97 (2.53)
General Disorders And Administration Site Conditions	Disease Progression	101	3.95 (3.25–4.81)	1.97 (1.69)	3.93 (221.02)	3.93 (3.34)
Infections And Infestations	Nasopharyngitis	95	2.39 (1.95–2.92)	1.25 (0.95)	2.38 (76.05)	2.38 (2.01)
Respiratory, Thoracic And Mediastinal Disorders	Respiratory Failure	88	5.46 (4.43–6.73)	2.44 (2.13)	5.43 (318.05)	5.42 (4.55)
Vascular Disorders	Poor Venous Access	86	39.55 (31.96–48.94)	5.28 (4.97)	39.3 (3,179.93)	38.94 (32.58)
Investigations	Oxygen Saturation Decreased	79	6.68 (5.35–8.34)	2.73 (2.41)	6.65 (378.77)	6.64 (5.52)
Injury, Poisoning And Procedural Complications	Infusion Related Reaction	75	5.39 (4.3–6.77)	2.42 (2.09)	5.37 (266.34)	5.36 (4.43)
Nervous System Disorders	Seizure	75	3.1 (2.47–3.89)	1.63 (1.29)	3.09 (106.14)	3.09 (2.55)
Respiratory, Thoracic And Mediastinal Disorders	Respiratory Distress	73	12.05 (9.57–15.17)	3.58 (3.24)	11.99 (733.21)	11.95 (9.86)
Cardiac Disorders	Cardiac Disorder	72	3.45 (2.73–4.34)	1.78 (1.44)	3.43 (124.19)	3.43 (2.83)
Cardiac Disorders	Tachycardia	72	3.7 (2.94–4.67)	1.88 (1.54)	3.69 (141.05)	3.68 (3.03)
General Disorders And Administration Site Conditions	Illness	72	3.98 (3.16–5.02)	1.99 (1.65)	3.97 (159.8)	3.96 (3.26)
Respiratory, Thoracic And Mediastinal Disorders	Respiratory Disorder	72	11.01 (8.73–13.88)	3.45 (3.11)	10.96 (649.97)	10.93 (9)
Infections And Infestations	Ear Infection	65	11.5 (9.01–14.68)	3.51 (3.16)	11.45 (618.41)	11.42 (9.31)
Nervous System Disorders	Hydrocephalus	60	50.47 (39.1–65.14)	5.63 (5.26)	50.24 (2,860.94)	49.65 (40.1)
Infections And Infestations	Device Related Infection	59	17.02 (13.18–22)	4.08 (3.7)	16.95 (882.31)	16.89 (13.63)

Abbreviations: SOC: system organ class; PT, preferred terms; ROR, reporting odds ratio; CI, confidence interval; PRR, proportional reporting ratio; χ2, chi-squared; IC, information component; IC025, the lower limit of 95%CI, of the IC; EBGM, empirical bayesian geometric mean; EBGM05, lower limit of 95% confidence interval of EBGM.

### 3.3 Sensitivity analysis

Among the 3,677 laronidase-related AEs retrieved from the FAERS database, 1,420 AEs (38.6%) lacked information on patient age. To mitigate the potential confounding effect of these age-unknown cases on the study results, a sensitivity analysis was performed. Following the exclusion of cases with unknown age, 2,198 valid reports remained for further analysis. Our results showed that after excluding the effect of unknown age, the common AEs were pyrexia, pneumonia, cough. This result suggests that there does not seem to be a large difference from the results of not excluding cases of unknown age (raw data in Supplementary Table S3).

### 3.4 Onset time of AEs

**FIGURE 4:**
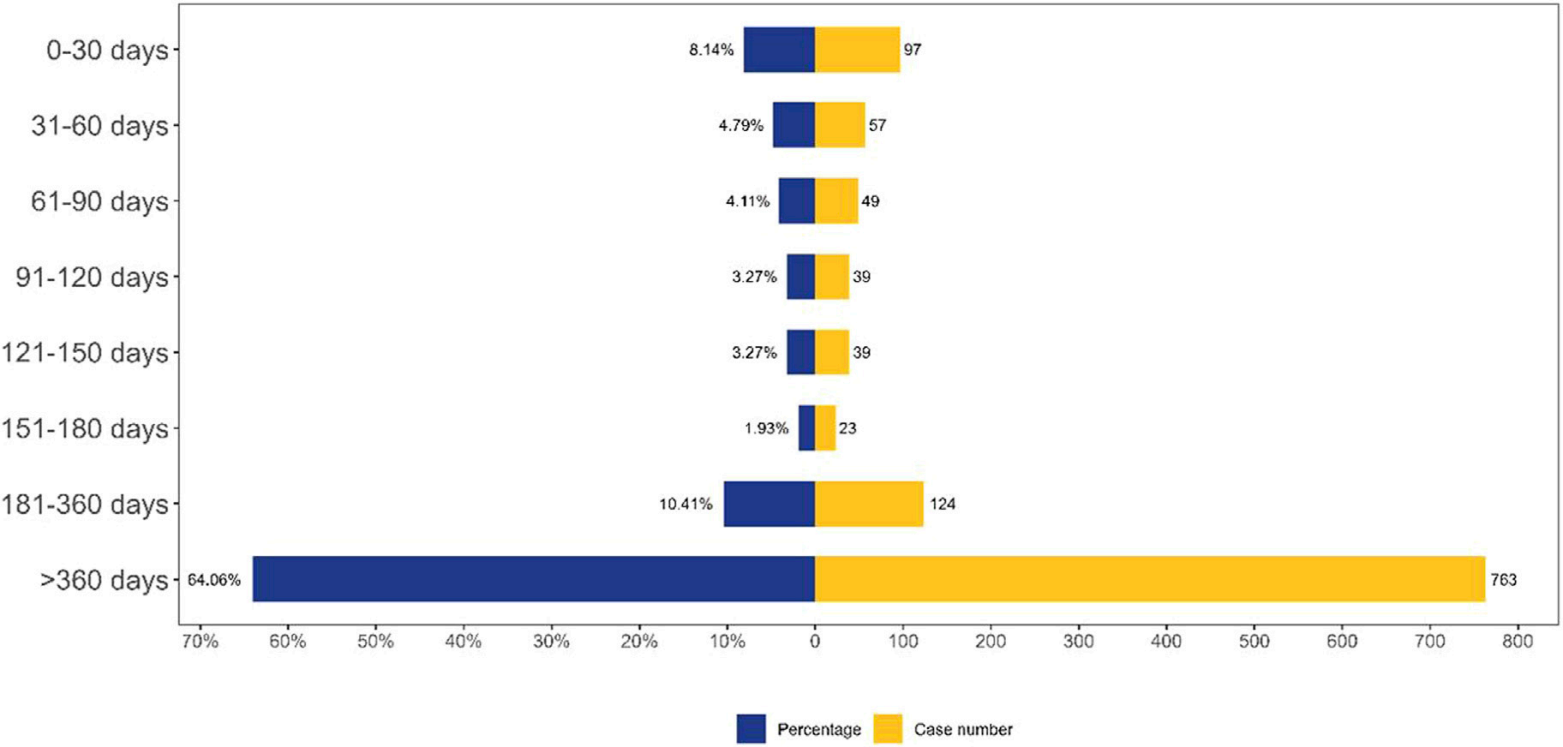
TTO analysis of laronidase -related AEs counted in days. Abbreviations: AE, adverse event.

In the FAERS database, a total of 1,191 records contained precise data on AE onset time. Figure 4 displayed the temporal distribution of AE occurrence, with the majority of cases (64.06%) reporting AE onset exceeding 360 days.

## 4 Discussion

Laronidase is a recombinant IDUA enzyme that catalyzes the hydrolysis of terminal α-L-iduronate residues in dermatan sulfate and heparan sulfate, thereby reducing systemic GAG accumulation (Machnikowska-Sokolowska et al., 2023; Zapolnik and Pyrkosz, 2022). In patients with MPS I, deficient IDUA activity leads to pathological GAG substrate deposition, resulting in widespread cellular, tissue, and organ dysfunction (Lipinski et al., 2024). Current therapeutic strategies include HSCT and ERT (Kubaski et al., 2020; Boelens et al., 2013). Following positive outcomes from a double-blind phase III clinical trial, laronidase received FDA approval as the first ERT agent for MPS I (2003).

This study described AE reports associated with laronidase from Q1 2004 to Q4 2024. Our analysis revealed a higher incidence of AEs in patients aged 5–18 years. This age-related pattern may be attributed to disease progression characteristics and diagnostic delays in MPS I. Patients with severe MPS I typically manifest symptoms within the first 6 months of life, and without treatment, they generally do not survive beyond the first decade (Beck et al., 2014). Individuals with the attenuated form of MPS I typically exhibit somatic manifestations in early childhood for the moderate-severe subtype, or in late childhood/adolescence for the mild subtype. Notably, those with the mild subtype can achieve a normal life expectancy with appropriate clinical management (Beck et al., 2014). A diagnostic history analysis demonstrated that 20% of patients with attenuated MPS I experienced diagnostic delays exceeding 5 years. These patients typically underwent consultations with four to five specialists sequentially before receiving an accurate diagnosis (Bruni et al., 2016). In a parallel cohort of 18 MPS I patients who manifested symptoms by 18 months of age, the mean age at biochemical confirmation was 75 months (Vieira et al., 2008). The results revealed that 64.06% of AEs emerged more than 360 days following treatment initiation, suggesting the need for systematic monitoring after the first year of therapeutic intervention.

The frequently reported AEs align with the labeled safety profile of laronidase, including pyrexia, pneumonia, cough, influenza, urticaria, disease progression, nasopharyngitis, respiratory failure, poor venous access, and decreased oxygen saturation. Consistent with this profile, a prospective open-label multinational study identified fever, diarrhea, vomiting, cough, rhinorrhea, rhinitis, and rash as the most common treatment-emergent AEs in all enrolled patients (Wraith et al., 2007). Similarly, a multinational, randomized, double-blind, placebo-controlled trial found that the most common adverse events in all enrolled patients were flushing, fever, headache, and rash (Wraith et al., 2004). In our PT analysis, pyrexia ranked first (n = 465), followed by pneumonia (n = 223) and cough (n = 167). The identified complications were significantly consistent with the AEs documented in previous laronidase clinical trials.

Fever emerged as the most prevalent treatment-related AE identified in our investigation. Consistent with other ERT regimens, laronidase administration was associated with the induction of anti-drug IgG antibodies (ADA) in more than 90% of patients during the initial therapeutic period (Giugliani et al., 2017). Preclinical evidence from canine models of MPS I demonstrated that ADA production compromised cellular uptake of the therapeutic enzyme in target tissues, resulting in impaired GAG catabolism, accompanied by elevated uGAG excretion levels (Dickson et al., 2008).Clinical observations further corroborated these findings, revealing a significant correlation between ADA seroconversion and attenuated pharmacodynamic responses, along with increased incidence of hypersensitivity reactions (Dickson et al., 2008). The meta-analysis indicated that the presence of ADA did not significantly impact clinical outcomes; however, 52% of patients experienced AEs potentially related to anaphylaxis (Xue et al., 2016). These findings suggest that ADA may constitute a primary factor in triggering hypersensitivity reactions among laronidase-treated patients, potentially explaining the observed fever, urticaria, and rhinitis manifestations. This evidence suggests that appropriate premedication regiments should be implemented prior to infusion to reduce the risk of life-threatening hypersensitivity reactions.

Among infectious and infective diseases, pneumonia, influenza, nasopharyngitis, ear infection, device-related infections, respiratory tract infection, viral infection, and upper/lower respiratory tract infections were high-frequency signals. These infection-related AEs were consistently documented in the product prescribing information. MPS I patients exhibit highly heterogeneous clinical manifestations, with symptoms progressing through diverse trajectories (Machnikowska-Sokolowska et al., 2023). The accumulation of unmetabolized GAG fragments triggers a cascade of secondary pathophysiological events, eventually leading to multi-system clinical manifestations including airway obstruction, restrictive lung disease, excessive viscous nasal secretions, and limited mouth opening (Murgasova et al., 2020). These anatomical and physiological alterations collectively heighten susceptibility to pathogenic microorganisms. The precise immunological mechanisms underlying infection predisposition in laronidase-treated patients remain incompletely characterized. Clinical investigations have demonstrated seroconversion rates exceeding 90% for anti-laronidase immunoglobulin G antibodies within the first 6 months of ERT initiation (Wraith et al., 2004). ADA titers typically exhibit temporal decline, with a subset of patients achieving seronegative conversion, indicating a possible immune tolerance development with sustained ERT (Wraith et al., 2004; Kakavanos et al., 2003). Specifically, frequent immunogenic responses may induce immune system dysregulation, potentially compromising innate defenses against pathogens and thereby increasing susceptibility to infection-related AEs during therapeutic intervention.

Cardiovascular involvement constitutes a prominent feature of MPS disorders and serves as a significant contributor to disease-related morbidity and mortality (Poswar et al., 2021). Given that GAGs are essential structural components of cardiac extracellular matrices, deficient degradation results in progressive lysosomal storage of undegraded GAGs within myocardial tissues (Poswar et al., 2021). This pathological accumulation may induce tissue damage through aberrant activation of cellular proteases, particularly matrix metalloproteinases, thereby disrupting cardiac architecture and function (Baldo et al., 2017; Poswar et al., 2021). Patients with MPS I exhibit heterogeneous cardiovascular manifestations, including valvulopathy, conduction system abnormalities, left ventricular hypertrophy, and accelerated coronary artery disease (Braunlin et al., 2011). Our results showed that tachycardia, cardiac arrest, acute heart failure cardiopulmonary arrest, progressive valvular disease, and mitral insufficiency were identified as high-frequency cardiac AEs related to laronidase treatment. This suggests that it is essential to monitor hemodynamics due to the inherent cardiovascular susceptibility of MPS I patients and the potential for recurrent infusion-related reactions. Furthermore, standardized cardiac function assessments, including echocardiography, serum B-type natriuretic peptide quantification, should be performed at baseline and every 6 months during therapeutic intervention.

Seizures and hydrocephalus emerged as noteworthy uncommon AEs in our investigation. Preclinical studies in MPS I murine models revealed significantly elevated cerebral GAG levels (up to 6-fold higher *versus* wild-type controls) (Eisengart et al., 2013). This pathological GAG deposition may impair cerebrospinal fluid reabsorption, leading to perivascular space enlargement, hydrocephalus, and ventriculomegaly (Hampe et al., 2021b). Central nervous system (CNS) manifestations, including structural abnormalities, are well-documented features of MPS. Structural brain imaging effectively demonstrates these CNS alterations in MPS patients (Luo et al., 2024). Quantitative volumetric studies further reveal widespread white matter involvement across the disease spectrum in MPS I (Shapiro et al., 2012). Beyond structural pathology, MPS significantly impacts CNS function, including neurocognitive deficits (Shapiro et al., 2012; Shapiro et al., 2015). Patients with severe neuropathic MPS subtypes commonly exhibit developmental delay, neurocognitive regression, behavioral disturbances, and sleep disorders (Shapiro et al., 2015; Hampe et al., 2021b; Luo et al., 2022).Seizure activity remains uncommon in MPS I patients, with documented cases predominantly observed following HSCT (Eisengart et al., 2013). This temporal association aligns with the well-established central nervous system morbidity profile characteristic of MPS disorders (Hampe et al., 2021b). However, the precise mechanisms underlying treatment-associated central nervous system pathophysiology remain incompletely elucidated. The limited penetration of intravenous ERT drugs across the blood-brain barrier indicates the urgent need for neurological monitoring during treatment (Schuh et al., 2024). Clinicians should be on high alert for emergent central nervous system-related manifestations and take timely neuroprotective measures when clinically suspected.

This study conducted pharmacovigilance analysis of laronidase-related AEs based on the FAERS database, however, several limitations were identified. As a self-reporting system, FAERS database relies on voluntary submissions, which introduces issues such as inconsistent data quality, reporting bias, underreporting and incomplete records. Non-professional reporters may overly emphasize common adverse events while neglecting rare events. The differences in pharmacovigilance in geographical regions also increase the risk of underreporting. Furthermore, the low prevalence of MPS I leads to a limited sample size, affecting the comprehensive analysis of AEs. In addition, the absence of original clinical records also impeded the analysis of key confounding factors. Therefore, clinicians should closely monitor clinical indicators after treatment and implement preventive measures to minimize complications and optimize therapeutic outcomes.

## 5 Conclusion

This study utilized the FAERS database to perform a comprehensive analysis of AEs associated with laronidase treatment, identifying both established and potential safety concerns. While laronidase has shown therapeutic efficacy for MPS I in clinical trials, our analysis revealed previously underrecognized AEs, including seizures and hydrocephalus. These results emphasized the necessity of monitoring and personalized treatment strategies during laronidase treatment. Furthermore, this study highlighted key research priorities, particularly investigating the pathophysiological mechanisms of laronidase-related AEs and optimizing management strategies to enhance treatment safety and clinical outcomes. These findings establish a foundation for advancing enzyme replacement therapies and refining clinical protocols in rare disease management.

## Data Availability

The original contributions presented in the study are included in the article/Supplementary Material, further inquiries can be directed to the corresponding authors.

## Abbreviations

MPS I, Mucopolysaccharidosis type I; IDUA, α-L-iduronidase; GAG, glycosaminoglycan; HSCT, hematopoietic stem cell transplantation; ERT, enzyme replacement therapy; FDA, Food and Drug Administration; uGAG, urinary glycosaminoglycan; AE, adverse event; FAERS, FDA Adverse Event Reporting System; PT, Preferred Term; MedDRA, Medical Dictionary for Regulatory Activities; SOC, System Organ Class; ROR, Reporting Odds Ratio; PRR, Proportional Reporting Ratio; BCPNN, Bayesian Confidence Propagation Neural Network; MGPS, Multi-Item Gamma Poisson Shrinker; ADA, anti-drug IgG antibodies; EBGM, Empirical Bayesian Geometric Mean.
